# “Yes, I’m interested in taking PrEP!”: PrEP interest among women respondents to the European community-based survey “Flash! PrEP in Europe”

**DOI:** 10.1371/journal.pone.0246037

**Published:** 2021-02-17

**Authors:** Rosemary M. Delabre, Adeline Bernier, Flor Sánchez, Antoine Vilotitch, Sophocles Chanos, Maria Luisa Cosmaro, Harriet Langanke, Coline Mey, Cary James, Sascha B. Duken, Vincent Schlegel, Richard Stranz, Kai J. Jonas, Bruno Spire, Daniela Rojas Castro

**Affiliations:** 1 Coalition PLUS, Community-Based Research Laboratory, Pantin, France; 2 Department of Social Psychology, Universidad Autónoma de Madrid, Madrid, Spain; 3 Aix Marseille Univ, INSERM, IRD, SESSTIM, Sciences Economiques & Sociales de la Santé & Traitement de l’Information Médicale, Marseille, France; 4 ORS PACA, Observatoire Régional de la Santé Provence-Alpes-Côte d’Azur, Marseille, France; 5 Ath & Thess Checkpoints, Athens, Greece; 6 Fondazione LILA Milano, Milan, Italy; 7 GSSG–Gemeinnützige Stiftung Sexualität und Gesundheit, Cologne, Germany; 8 AIDES, Pantin, France; 9 Terrence Higgins Trust, London, United Kingdom; 10 University of Amsterdam, Amsterdam, The Netherlands; 11 Maastricht University, Maastricht, The Netherlands; Centers for Disease Control and Prevention, UNITED STATES

## Abstract

The World Health Organization recommends pre-exposure prophylaxis (PrEP) for all populations at substantial risk of HIV infection, including women. However, data regarding PrEP interest among women is lacking, particularly in Europe. Factors associated with interest in using PrEP were assessed among women respondents to the Flash! PrEP in Europe (FPIE) survey. This community-based cross-sectional study, conducted in 12 European countries, aimed to assess PrEP knowledge and interest. “High objective risk” (HOR) was assessed using established risk criteria following EACS and CDC guidelines. Factors associated with interest in using PrEP were assessed in univariable and multivariable logistic regression models. Among 678 women, 12.5% (n = 85) were considered at HOR, 46.8% (n = 317) indicated prior PrEP knowledge and 18.0% (n = 122) reported interest in using PrEP. Among women at HOR, 40.0% (n = 34) were interested in PrEP. Factors significantly associated with PrEP interest in the final multivariable model were: younger age (18–29 years) (aOR 1.91[95CI: 1.07; 3.41]), bad self-perceived financial status (1.84[1.09; 3.11]), migrant status (south to north) (2.87[1.05; 7.89]), single or dating relationship status (1.93[1.23; 3.03]), sexual abuse history (1.86[1.17; 2.97]), “rather high”/ “high” self-perceived HIV risk (3.21[1.32; 7.81]), and HOR (2.49[1.42; 4.35]). These results show that women at HOR and those who perceived themselves to be at high risk are interested in using PrEP. There is a critical need for targeted information and improved access to PrEP to increase uptake of this HIV prevention tool to meet PrEP interest among women.

## Introduction

Promotion of available and development of new HIV prevention tools adapted to the needs of women and young adolescent girls are crucial to lowering the burden of HIV among this group. In Europe, the rate of new HIV diagnoses among women is lower compared to men; however, heterosexual contact is the main mode of HIV transmission in certain countries [1]. Due to various biological, social and cultural factors, women and adolescent girls are particularly at risk for HIV infection [2–4]. Furthermore, HIV prevalence may be higher in specific groups of women, such as sex workers [5] and women who have experienced intimate partner violence [6–8]. Gender inequality has largely been considered a major driving force of the HIV epidemic in the context of heterosexual transmission [4, 9, 10].

Recognition of the gender-related specificities of the HIV epidemic has led to a call for the development of comprehensive HIV prevention programs which strengthen women’s capacities on sexual and HIV risk, and empower them to protect themselves; community involvement and support are essential elements of these programs [3, 9]. Oral pre-exposure prophylaxis (PrEP), which is the use of antiretroviral drugs by HIV-negative individuals to reduce the risk of HIV infection, may be an important self-controlled prevention method for women. Efficacy of daily oral tenofovir disoproxil fumarate/emtricitabine (TDF/FTC) as PrEP has been shown in randomized controlled trials among different populations (heterosexual men and women) [11, 12]. However, other studies evaluating PrEP efficacy and involving only women have found no associated reduction in risk [13, 14]. The lower efficacy observed in these studies has been attributed, in part, to adherence issues linked to various factors such as an underestimation of personal risk of HIV infection, PrEP or HIV-related stigma from the community and fear of side effects [15–18]. Despite a variance in levels of efficacy among women, PrEP is still considered a viable tool for self-controlled HIV prevention for women.

The World Health Organization (WHO) recommended PrEP for HIV prevention among men who have sex with men (MSM) in 2014 [19] and expanded their recommendation to “all population groups at substantial risk of HIV infection” in 2015 [20]. European guidelines on PrEP offer more detailed recommendations for MSM and transgender people but add that PrEP “may be considered” for HIV-negative heterosexual men and women who do not consistently use condoms or who have potentially untreated HIV-positive partners [21]. Although these broader recommendations theoretically allow more women to access and benefit from PrEP, uptake of PrEP among women remains largely dependent on knowledge, personal HIV risk assessment and interest [22].

Currently, much of the research on PrEP knowledge and potential uptake among (primarily at risk) women has taken place in the United States (US) and in some African countries [22–27]. Information regarding PrEP knowledge and interest among women is severely lacking within Europe, where PrEP access is still limited. With more European countries authorizing the prescription and reimbursement of PrEP, it is crucial to collect standardized data regarding PrEP, to ensure public health policies are engaging, responding and supportive of the needs of women. The objective of this analysis was to identify factors associated with interest in using PrEP among women respondents to a large, European community-based survey.

## Materials and methods

### Study organization and study population

The Flash! PrEP in Europe (FPIE) survey was a community-based cross-sectional study aiming to assess knowledge of, attitudes towards, interest to use and current use of PrEP. FPIE was conducted with the University of Amsterdam and the Universidad Autónoma de Madrid, as well as 15 non-governmental organizations (NGOs) based in 12 European countries (Denmark, France, Germany, Greece, Ireland, Italy, the Netherlands, Portugal, Romania, Spain, Switzerland, and the United Kingdom). People living with, exposed to HIV and/or involved in the fight against HIV actively participated throughout the entire project. This research was conducted as a part of “Flash PrEP in Europe,” a joint European research project, coordinated by the community-based NGOs AIDES and Coalition PLUS, in partnership with the University of Amsterdam. Participating NGOs (see acknowledgements) were identified through an informal network or word of mouth and dedicated their own resources to the survey. NGOs were actively involved in the questionnaire conception, validation, and translation. NGOs also promoted the survey, in addition to participating alongside experts and academics in the steering and scientific committees during which results and analyses were discussed.

The 82-item, self-completed, anonymous and voluntary online questionnaire was available from 15 June to 15 July 2016 in ten languages and diffused in the participating countries. At the time the survey was conducted, PrEP was only officially available and reimbursed in France. The Ethics Review Board of the Faculty of Social and Behavioral Sciences, University of Amsterdam, the Netherlands, granted approval for the study (2016-SP-7030). Eligible respondents were at least 18 years old and declared being HIV negative or unaware of their serological status. Respondents who were HIV positive or who did not wish to report their HIV status were excluded. All respondents provided written informed consent before starting the questionnaire.

### Survey promotion/recruitment

A convenience sampling method was used with a specific focus on all populations highly exposed to HIV. Study promotion aimed to target all key populations on the European and country level, including: migrants (especially those from endemic African countries), people who use drugs, HIV-negative members within serodifferent relationships, trans people, and people who engage(d) in transactional sex. Women were therefore not specifically targeted as a group in itself, but rather would be concerned based on whether they identified with one of the groups above.

All participating organizations were encouraged to communicate about the survey on their social media pages, during regular activities, and to work with other organizations that would be willing to promote the survey. Promotion methods used by NGOs included internal mail listings, websites, social media, organization website, as well as dating apps/websites. European organization such as the European AIDS Treatment Group (EATG)), the International Lesbian, Gay, Trans and Intersex association (ILGA) diffused the link for the survey. Informative websites including Aidsmap/NAM, PrEPster and PrEP Watch also diffused messages about the survey on their websites. Banner ads were also displayed on dating applications and websites Hornet® and PlanetRomeo®. See S1 File for more information.

### Survey instrument

PrEP was briefly described at the start of the survey as a new prevention tool against HIV and the respondents were informed of the countries where it was currently authorized. Respondents were then asked if they knew of PrEP before the survey, and were subsequently given a more complete, but brief, explanation of PrEP. The questionnaire is found in the S2 File.

Prior knowledge of PrEP, interest in using PrEP and current use of PrEP were assessed. Data related to socio-demographic characteristics (including sex at birth, current gender, age, country of birth, current country of residence, education level, and perceived financial situation) were collected in addition to information on sexual activity in the last 6 months, number and gender of sexual partners, condom use, history of sexual abuse, history of transactional sex, drug taking behaviors, frequency of HIV and other sexually transmitted infections (STI) testing, number of STI diagnoses, and perceived HIV and STI risk.

### Data and analysis

Respondents included in this analysis declared female sex at birth and at the time of the survey. Respondents with a migrant background were defined as those who were born in a country different from where they currently lived; region of birth and current residence (North or South) was determined based upon the Brandt line as defined in the Brandt report [28]. Respondents who reported using condoms “never”, “rarely”, “from time to time” or “nearly always” in the past 6 months with their occasional sex partners (excluding main sex partner if they had one) were considered to have inconsistent condom use compared to those that declared having “always” used condoms. Sexual abuse was defined as “sex against [one’s] will because of verbal, physical or any other form of pressure”. Transactional sex was defined as “money, goods or drugs in exchange for sex”. Respondents indicated their HIV risk perception using a 5-point Likert scale, ranging from 1 for low to 5 for high; this variable was recategorized as “low/rather low”, “average”, or “rather high/high”.

A variable was created to identify women who would be considered at high objective risk (HOR) for HIV infection following a selection of EACS and CDC guidelines [21, 29, 30]. The following criteria were used: (i) two or more occasional male sex partners in the previous 6 months and inconsistent condom use during vaginal or anal sex in the previous 6 months; or (ii) two or more STI diagnoses in the previous twelve months; or (iii) drug injection in a sexual context in the previous twelve months; or (iv) seropositive main sex partner with a detectable or unknown viral load.

Interest in using PrEP was the primary outcome studied. Interest in using PrEP was assessed with the question: “Are you interested in using PrEP?”; respondents who replied “No, definitely not”, “No, probably not” or “Maybe” were compared to those who replied “Yes, probably” or “Yes, definitely”. Differences between women who were interested in PrEP compared to those who were maybe/not interested were examined using the chi-square or Fisher test for categorical variables. Factors associated with interest in using PrEP were assessed in univariable and multivariable logistic regression models. Variables for which p<0.20 in univariable analysis were included in the multivariable analysis. The multivariable model was built using a complete case approach. A backward procedure based on the Likelihood Ratio Chi-2 test was used to select significant variables for the final model (p-value <0.05).

Qualtrics® software [31] was used in the development of the survey and data collection. Statistical analyses were conducted using STATA (12.1) and SPSS (version 20.0.0).

## Results and discussion

A total of 15 880 individuals responded to the FPIE survey, of whom 907 (5.7%) were women. Romanian women respondents (n = 217) were excluded from the analysis due to a translation error. PrEP interest information was missing for 6 women and another 6 were using PrEP at the time of the survey; these 12 women were, thus, excluded from the current analysis.

**Fig 1:**
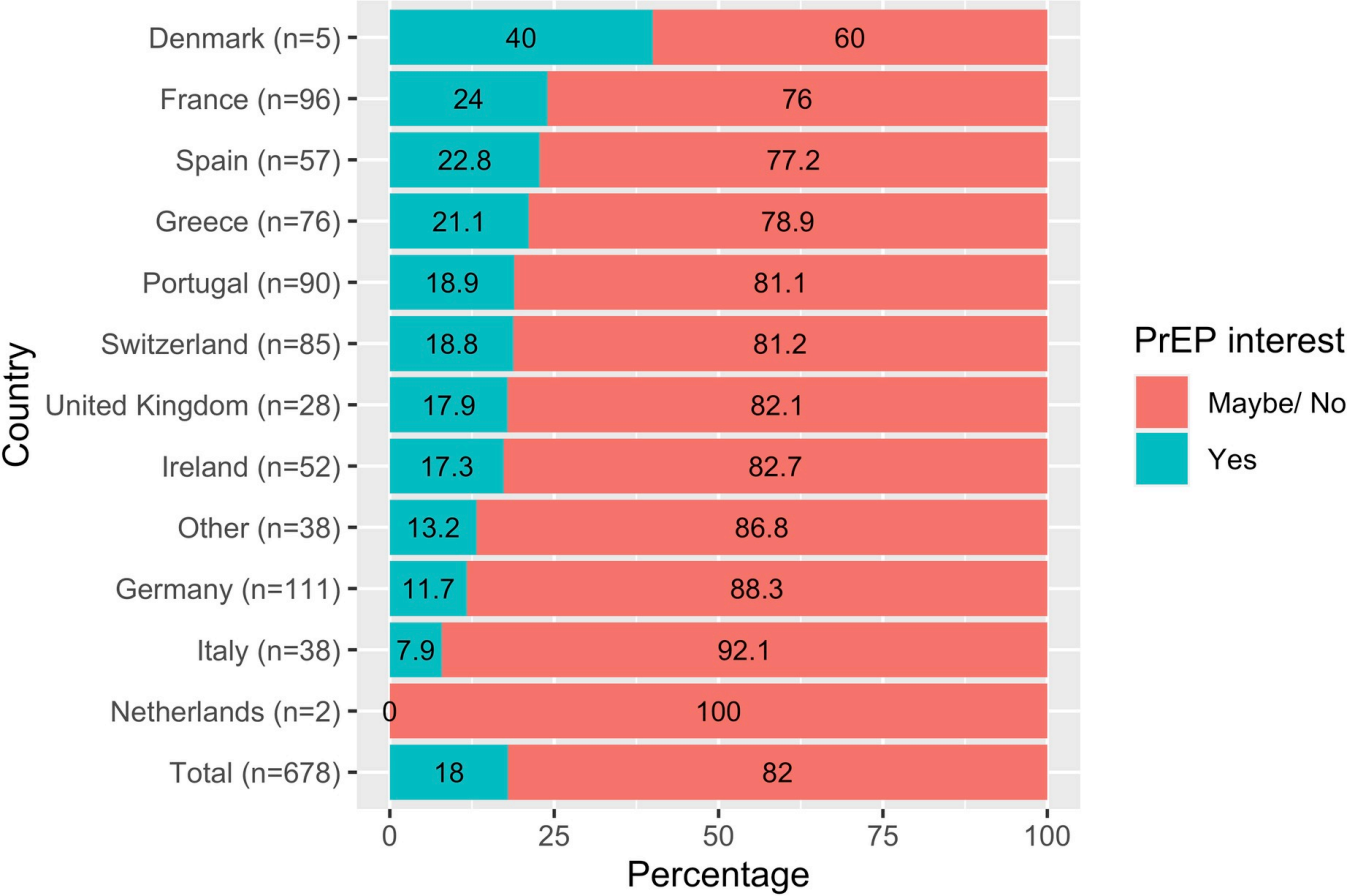
PrEP interest among women respondents to the Flash! PrEP in Europe survey by participating country.

The 678 women included in this analysis resided in 11 different European countries participating in the survey; the majority came from Germany (n = 111, 16.4%), France (n = 96, 14.2%), Portugal (n = 90, 13.3%) and Switzerland (n = 85, 12.5%). Thirty-eight women (5.6%) resided in other countries: 26 resided in other European countries and 12 were residing in countries outside of Europe. Almost half 46.8% (n = 317) knew of PrEP prior to the survey. Regarding PrEP interest, 122 women (18.0%) declared that they “probably” or “definitely” would be interested in using PrEP. PrEP interest varied by country (Fig 1) ranging from 0.0% (in the Netherlands) to 40.0% (in Denmark), however these percentages should be interpreted with caution due to small sample sizes in both countries.

Characteristics of the study sample according to PrEP interest are presented in Table 1. Almost half of the women included in the analysis (48.9%, n = 331) were between 18 and 29 years old and 50.9% (n = 345) lived in a city (population of more than 500 000). A large majority (77.8%, n = 523) had a bachelor’s degree or higher and 83.8% (n = 568) reported fair/good perceived financial situation. Close to one fifth of the women (17.5%, n = 118) were migrants, primarily coming from other northern countries. Sixty-one percent (n = 415) were in a relationship and 25.8% (n = 175) had at least one child. Almost three-quarters 72.5%, (n = 490) were satisfied with their life in general. Regarding sexual activity, 77.1% (n = 523) had sex within the last 6 months, 70.8% (n = 478) had sex with men and 73.0% (n = 420) had a main sexual partner. One quarter of women 24.8%, (n = 161) reported history of sexual abuse, 4.8% (n = 31) reported history of transactional sex, and 8.0% (n = 53) have used non-injection drugs in a sexual context. Thirty percent (n = 203) had during the past 12 months at least one HIV recent test and 4.3% (n = 28) were diagnosed with an STI. A small proportion 4.0% (n = 27) evaluated their risk of becoming infected with HIV as “rather high” or “high”, however, eighty-five women (12.5%) were considered at HOR for HIV according to the defined criteria. A large majority of women at HOR (90.6%, n = 77) met the criteria regarding two or more occasional male sex partners in the previous 6 months and inconsistent condom use during vaginal or anal sex in the previous 6 months. Among women identified at HOR, 40.0% (n = 34) were interested in PrEP.

**Table 1:** Main characteristics of women respondents to the FPIE survey according to interest in PrEP (n = 678).

	Interest in using PrEP			
	No/Maybe (n = 556; 82%), n(%)	Yes (n = 122; 18%), n(%)	Total (n = 678), n(%)	*P*-value
Age				**0.002**
• 18–29 years	254(45.7)	77(63.6)	331(48.9)	
• 30–39 years	171(30.8)	24(19.8)	195(28.8)	
• 40 years and more	131(23.6)	20(16.5)	151(22.3)	
Population size of place of residence				0.701
• Less than 500.000 inhabitants	275(49.5)	58(47.5)	333(49.1)	
• More than 500.000 inhabitants	281(50.5)	64(52.5)	345(50.9)	
Education level (current or highest obtained)				**0.027**
• Lower than Bachelor	113(20.5)	36(29.8)	149(22.2)	
• Bachelor or higher	438(79.5)	85(70.2)	523(77.8)	
Self-perceived financial situation				**0.006**
• Bad	80(14.4)	30(24.6)	110(16.2)	
• Fair/Good	476(85.6)	92(75.4)	568(83.8)	
Migrant (born in a different country)				**0.034**
• No	462(83.4)	96(78.7)	558(82.5)	
• From North to North	80(14.4)	18(14.8)	98(14.5)	
• From South to North	12(2.2)	8(6.6)	20(3.0)	
Children				**0.004**
• No	400(71.9)	103(84.4)	503(74.2)	
• Yes	156(28.1)	19(15.6)	175(25.8)	
Current relationship situation				**<0.001**
• Single/ Dating	196(35.3)	67(54.9)	263(38.8)	
• In a relationship (including open relationship)	360(64.7)	55(45.1)	415(61.2)	
Satisfaction in life in general				0.064
• Dissatisfied	73(13.2)	22(18.0)	95(14.1)	
• Neither satisfied or dissatisfied	69(12.5)	22(18.0)	91(13.5)	
• Satisfied	412(74.4)	78(63.9)	490(72.5)	
Ever had sex				0.458
• No, never	19(3.4)	7(5.7)	26(3.8)	
• Yes, more than 6 months	105(18.9)	24(19.7)	129(19.0)	
• Yes, in the past 6 months	432(77.7)	91(74.6)	523(77.1)	
Sexual activity in past 6 months				0.663
• No activity	124(22.4)	31(25.4)	155(23.0)	
• With men	393(71.1)	85(69.7)	478(70.8)	
• Only with women or trans	36(6.5)	6(4.9)	42(6.2)	
Currently have a main sexual partner				0.057
• No	124(25.5)	31(35.2)	155(27.0)	
• Yes	363(74.5)	57(64.8)	420(73.0)	
Number of occasional male sex partners in the previous six months				**0.514**
• 0	2 (1.6)	0 (0.0)	2 (1.2)	
• 1	34 (27.4)	11 (22.4)	45 (26.0)	
• 2 or more	88 (71.0)	38 (77.6)	126 (72.8)	
Inconsistent condom use during vaginal or anal sex in the previous 6 months				**0.001**
• No	61 (50.4)	10 (20.4)	71 (41.8)	
• Yes	60 (49.6)	39 (79.6)	99 (58.2)	
History of sexual abuse				**0.001**
• No	417(77.9)	72(62.6)	489(75.2)	
• Yes	118(22.1)	43(37.4)	161(24.8)	
History of transactional sex				0.227
• No	511(95.7)	107(93.0)	618(95.2)	
• Yes	23(4.3)	8(7.0)	31(4.8)	
Drug use (other than by injection)				0.679
• No, never	266(47.8)	61(50.0)	327(48.2)	
• Yes, more than 12 months ago	149(26.8)	28(23.0)	177(26.1)	
• Yes, in the past 12 months	141(25.4)	33(27.0)	174(25.7)	
Chem sex (other than by injection)				0.615
• No	502(92.3)	110(90.9)	612(92.0)	
• Yes	42(7.7)	11(9.1)	53(8.0)	
Number of HIV tests in the past year				**0.008**
• 0	403(72.6)	71(58.2)	474(70.0)	
• 1	111(20.0)	34(27.9)	145(21.4)	
• 2	29(5.2)	10(8.2)	39(5.8)	
• 3 or more	12(2.2)	7(5.7)	19(2.8)	
Diagnosed for an STI in the past year				**0.040**
• No	517(96.5)	106(92.2)	623(95.7)	
• Yes	19(3.5)	9(7.8)	28(4.3)	
Self-perceived risk of becoming infected with HIV				**<0.001**
• Low/Rather low	494(88.8)	89(73.0)	583(86.0)	
• Average	48(8.6)	20(16.4)	68(10.0)	
• Rather high/High	14(2.5)	13(10.7)	27(4.0)	
High objective risk for HIV				**<0.001**
• No	505(90.8)	88(72.1)	593(87.5)	
• Yes	51(9.2)	34(27.9)	85(12.5)	
Prior knowledge of PrEP				0.158
• No	289(52.0)	72(59.0)	361(53.2)	
• Yes	267(48.0)	50(41.0)	317(46.8)	

Results of the univariable and multivariable analyses are presented in Table 2. The univariable analyses identified several factors that were significantly associated with interest in using PrEP: age, education level, perceived financial status, migrant status, (having) children, relationship status, history of sexual abuse, number of HIV tests in the past year, STI diagnosis in the past year, perceived HIV risk, and HOR status. In the final multivariable model, seven variables were associated with higher interest in using PrEP: younger age (18–29 years old) (aOR: 1.91, 95% CI: 1.07–3.41), “bad” self-perceived financial status (1.84[1.09–3.11)], migrant status (South to North) (2.87[1.05–7.89]), not being in a relationship (single or dating) (1.93[1.23–3.03]), sexual abuse history (1.86[1.17–2.97]), having “rather high”/ “high” self-perceived risk of becoming infected with HIV (3.21[1.32–7.81]), and high objective HIV risk status (2.49[1.42–4.35]).

**Table 2:** Factors associated with PrEP interest–univariable (n = 678) and multivariable (n = 649) logistic regression models.

	Univariable model (n = 678)		Multivariable model (n = 649)	
	OR[95%CI]*	*P*-value	aOR[95%CI]*	*P*-value
Age				
• 18–29 years	**1.99[1.16; 3.39]**	**0.012**	**1.91[1.07; 3.41]**	**0.028**
• 30–39 years	0.92[0.49; 1.74]	0.795	0.94[0.48; 1.84]	0.851
• 40 years and older	Ref			
Population size of place of residence				
• Less than 500 000 inhabitants	0.93[0.63; 1.37]	0.701		
• More than 500 000 inhabitants	Ref			
Education level				
• Lower than Bachelor	Ref			
• Bachelor or higher	**0.61[0.39; 0.95]**	**0.028**		
Self-perceived financial situation				
• Bad	**1.94[1.21; 3.12]**	**0.006**	**1.84[1.09; 3.11]**	**0.023**
• Fair/Good	Ref		Ref	
Migrant (born in a different country)				
• No	Ref		Ref	
• From North to North	1.08[0.62; 1.89]	0.779	0.85[0.44; 1.64]	0.629
• From South to North	**3.21[1.28; 8.06]**	**0.013**	**2.87[1.05; 7.89]**	**0.041**
Children				
• No	Ref			
• Yes	**0.47[0.28; 0.80]**	**0.005**		
Current relationship situation				
• Single/ Dating	**2.24[1.50; 3.33]**	**<0.001**	**1.93[1.23; 3.03]**	**0.004**
• In a relationship (including open)	Ref		Ref	
Satisfaction in life in general				
• Dissatisfied	Ref			
• Neither satisfied or dissatisfied	1.06[0.54; 2.08]	0.870		
• Satisfied	0.63[0.37; 1.07]	0.088		
Ever had sex				
• No, never	1.75[0.71; 4.28]	0.221		
• Yes, more than 6 months	1.09[0.66; 1.78]	0.748		
• Yes, in the past 6 months	Ref			
Sexual activity in the past 6 months	Ref			
• No activity	0.87[0.55; 1.37]	0.535		
• With men	0.67[0.26; 1.72]	0.403		
• Only with women or trans				
Currently have a sexual main partner	Ref			
• No	0.63[0.39; 1.02]	0.059		
• Yes				
History of sexual abuse				
• No	Ref		Ref	
• Yes	**2.11[1.37; 3.24]**	**0.001**	**1.86[1.17;2.97]**	**0.009**
Transactional sex				
• No, never	Ref			
• Yes	1.66[0.72; 3.81]	0.231		
Drug use (not by injection)				
• Yes, in the past 12 months	1.02[0.64; 1.63]	0.932		
• Yes, more than 12 months	0.82[0.50; 1.34]	0.426		
• No, never	Ref			
Chem sex (drug use in sexual context)	Ref			
• No	1.20[0.60; 2.40]	0.615		
• Yes				
Number of HIV tests in the past year				
• 0	Ref			
• 1	**1.74[1.10; 2.75]**	**0.018**		
• 2	1.96[0.91; 4.19]	0.084		
• 3 or more	**3.31[1.26; 8.70]**	**0.015**		
Diagnosed with an STI in the past year				
• Yes	**2.31[1.02; 5.25]**	**0.045**		
• No	Ref			
Self-perceived risk of becoming infected by HIV				
• Low/Rather low	Ref		Ref	
• Average	**2.31[1.31; 4.08]**	**0.004**	1.54[0.81; 2.90]	0.185
• Rather high/High	**5.15[2.34; 11.33]**	**<0.001**	**3.21[1.32; 7.81]**	**0.010**
High objective risk for HIV				
• No	Ref		Ref	
• Yes	**3.83[2.35; 6.24]**	**<0.001**	**2.49[1.42; 4.35]**	**0.001**
Prior knowledge of PrEP				
• No	Ref			
• Yes	0.75[0.51; 1.12]	0.159		

*CI: Confidence Interval.

This analysis of women respondents of a large European survey brings valuable information regarding self-perceived HIV risk assessment, PrEP knowledge and interest that is crucial to informing effective public health prevention programs for women in the context of increasing availability of PrEP in Europe. Close to half of the women respondents had prior PrEP knowledge but interest in using PrEP was lower. Importantly, women who had high subjective and objective HIV risk showed a vested interest in PrEP. Additionally, results show an association between social determinants of health (age, socio-economic status, migrant status) and PrEP interest which further highlight the need to address structural factors that have an impact on HIV risk and adherence to PrEP.

Women identified at HOR and who are interested in using PrEP are ideal targets for PrEP implementation programs and identification of these women is vital. A total of 85 (12.5%) women in this analysis would be considered at HOR for HIV, and according to the results of the multivariable model, the odds of being interested in PrEP among this group of women were 2.49 times that of women who were not identified at HOR. HIV risk assessment tools have been developed [32] in specific subgroups of women such as African women [33], serodifferent couples [34, 35], and pregnant and postpartum women [36], and are increasingly important to help women understand their (and their partner’s) HIV risk. For medical providers, these and other tools such as algorithms that can identify a patient’s risk based on their medical data [37], may be helpful to identify those who may underestimate their risk [22] and who may benefit from PrEP.

In this analysis, women with rather high/high HIV risk perception also reported higher interest in PrEP, supporting previous findings among women [32, 38, 39] and other key populations, that perception of HIV risk is an important factor of PrEP interest [40–43]. Additionally, high risk perception may play an important role in PrEP adherence [17, 39, 44], a key lesson from the PrEP implementation process in the US [40]. It is important to underline, however, that a majority of women in this sample who were considered at HOR for HIV were not interested in using PrEP (51 of 85 or 60%). Furthermore, 63% (n = 32) evaluated their HIV risk to be “low” or “rather low”. These results suggest that more work needs to be done to empower women to better evaluate their risk of HIV and to identify and implement pertinent risk reduction strategies.

In this study, younger age and a financial situation perceived to be bad were associated with higher interest in using PrEP. The association between low socio-economic status and increased likelihood of reporting potential PrEP uptake has been previously been reported [45]. Several studies have identified the need to integrate structural interventions in prevention programs to address social determinants which contribute to HIV risk among women [46–49] and other at-risk groups [50]. The association between history of sexual or physical gender-based violence and engagement in HIV-related risk behavior has been well established in various settings and particularly among female sex workers [51–54]. PrEP could be an important method for empowering at-risk women [38, 55], particularly those who are affected by synergistic epidemics of substance abuse, violence, and HIV/AIDS and who are otherwise at a disadvantage with regard to condom negotiation [49, 56]. Increasing knowledge and interest in using PrEP, while facilitating its authorization and access, is critical for expanding PrEP uptake among women. However, effective prevention strategies should not rely on this biomedical intervention alone and should address structural factors that are beyond the scope of individual choice and behavior for a maximal effect at the population level [50, 57].

Study results regarding PrEP knowledge and interest differ from other studies among women and other at risk groups which generally show low prior PrEP knowledge, but rather high interest in PrEP after receiving information on it [24, 58, 59]. In contrast to a majority of studies investigating PrEP, which focused on female at risk populations such as sex workers, African-Americans (US studies), and areas of high HIV prevalence [23, 45, 60–62], the FPIE survey reached a broader population of women in an understudied region (Europe). Women in this study had a higher level of knowledge compared to other studies based on at risk women in the US in which PrEP knowledge ranged from almost 0 to 33% [23, 61, 63]. This result may be a consequence of the population sampled here, who may be more connected to HIV prevention services and organizations [64]. Regarding PrEP interest, women in this study expressed relatively low (18.0%) interest, which is comparable to another US study citing only 20% willingness to use PrEP among Caribbean immigrant women, but contrasts with other reports of high (60–65%) interest among at risk women in the US and internationally [23, 60, 61, 65].

### Limitations

Due to the methodology used for this survey (promotion of the survey by NGOs, on social network and/or dating applications/websites, online questionnaire) respondents were not representative of European women and therefore results may not be generalizable; yet, this is one of the first data sets on this population. No information was collected for the individuals who refused study participation and therefore we are unable to assess if they are significantly different from respondents. The possibility of social desirability bias regarding sensitive items cannot be excluded. While the survey was conducted in 2016, current data shows PrEP is only fully reimbursed and available in 16 of 49 reporting European and Central Asian countries [66]. PrEP interest was assessed based on hypothetical use at the time of assessment however, according to recent data [66], PrEP uptake is overwhelmingly concentrated among MSM. Finally, as PrEP availability increases in Europe, future studies will be able to evaluate the determinants of actual PrEP uptake.

## Conclusion

While overall interest in using PrEP was low, women in Europe who are at high objective HIV risk and those who perceive themselves to be at high HIV risk are interested in using PrEP. In addition to increasing knowledge on PrEP among women, it is critical that efforts are made on the national level to explicitly include women in the national guidelines and to develop PrEP services that meet their specific needs. Community-based approaches and interventions may be particularly relevant to reach at risk women and help improve prevention package access. Finally, there is a need for structural approaches in HIV prevention, to tackle underlying mechanisms of gender-based inequalities.

## Supporting information

S1 File Flash! PrEP in Europe survey promotion. (DOCX)

S2 File Flash! PrEP in Europe survey, English version. (DOCX)

S1 Dataset Flash! PrEP in Europe dataset for analysis. (DTA)
