# Supplementary material for: “Yes, I’m interested in taking PrEP!”: PrEP interest among women respondents to the European community-based survey “Flash! PrEP in Europe”
Source: PLoS One. 2021 Feb 17;16(2):e0246037. doi: 10.1371/journal.pone.0246037 (PMC7888674; doi:10.1371/journal.pone.0246037)
Supplement: S1 File — (DOCX) [file pone.0246037.s001.docx]

# **S1 Supporting information. Flash! PrEP in Europe survey promotion.**

General guidelines concerning the communication strategy was provided to all organizations to widely disseminate the survey and ensure a large number of respondents. All organizations were encouraged to communicate about the survey on their social media pages, during regular activities, and to work with other organizations that would be willing to promote the survey as well as towards target populations.

All populations highly exposed to HIV were considered as potential targets for the survey: MSM, migrants (especially those from endemic African countries), people who use drugs, HIV-negative individuals within serodifferent relationships, trans people, and people who engage(d) in transactional sex. Women were therefore not specifically targeted as a group in itself, but rather would be concerned based on whether they identified with one of the groups above.

Specifically, the survey was promoted both on an European and national level. On the European level, international organizations (such as the European AIDS Treatment Group (EATG)) actively promoted the survey on its website and social media pages or the International Lesbian, Gay, Trans and Intersex association (ILGA) spread the link for the survey through their internal mailing list. Informative websites including Aidsmap/NAM, PrEPster and PrEP Watch also diffused messages about the survey on their websites. Promotion on dating applications and websites was also conducted. The dating app/website Hornet® agreed to promote the survey across Europe throughout the data collection period. Banner and native ads (in English) were displayed to all users according to their operating system. PlanetRomeo® also displayed banners translated in 10 languages in all participating countries, apart from Germany.

Regarding national promotion, each participating NGO was provided with 2 000 cards to promote the survey during their regular activities (gay prides, rapid HIV testing etc.). The cards only presented the logo, the link to the survey and a QR code. These communication tools were supposed to help them to get hard-to-reach subpopulations, such as women, people who engage(d) in transactional sex, migrants, people who use drugs or trans people. Additionally, electronic versions of flyers/ posters were translated into 10 languages. Translated business cards and online banners were also provided to participating NGOs.
