# Supplementary material for: “Yes, I’m interested in taking PrEP!”: PrEP interest among women respondents to the European community-based survey “Flash! PrEP in Europe”
Source: PLoS One. 2021 Feb 17;16(2):e0246037. doi: 10.1371/journal.pone.0246037 (PMC7888674; doi:10.1371/journal.pone.0246037)
Supplement: S2 File — (DOCX) [file pone.0246037.s002.docx]

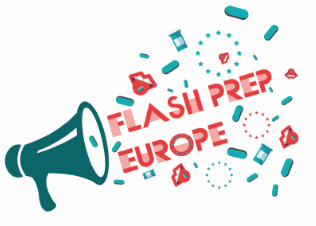
**S2 File. Flash! PrEP in Europe survey, English version.**

|  |  |
| --- | --- |

**English Version of the 2016 EU Flash PrEP Survey**

For scientific information please contact:

Dr. Daniela Rojas Castro

drojascastro@coalitionplus.org

Dr. Kai Jonas

[kai.jonas@maastrichtuniversity.nl](mailto:kai.jonas@maastrichtuniversity.nl)


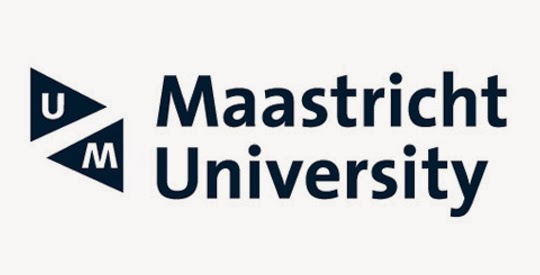

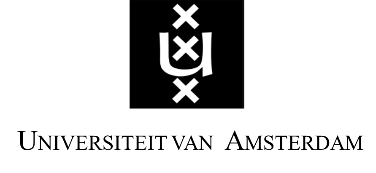

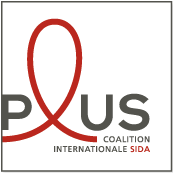

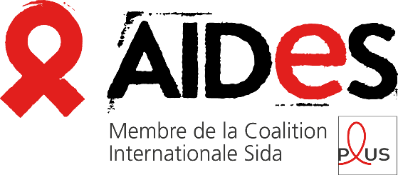
age_check I agree to the above conditions and confirm that I am 18 years or older

- I am 18 years or older and I want to participate in the survey (1)
- I am younger than 18, or I do not want to participate in the survey (2)

Display This Question:

If I agree to the above conditions and confirm that I am 18 year and older I am younger than 18, or I do not want to participate in the survey Is Selected

END_1 Sorry, for ethical reasons this survey is open to people over the age of 18 who wish to participate. If you need further information, please contact us at: XXXXX

If This survey is only intende... Is Displayed, Then Skip To End of Survey

HIVstat Before we start with the survey, we would like to know your HIV status.

- I'm HIV-positive (1)
- I'm HIV-negative (2)
- I don't know my HIV status (3)
- I don't want to provide this information (4)

Display This Question:

If What is your HIV serological status ? I'm HIV-positive Is Selected

pos PrEP refers to the use of antiretroviral drugs to prevent HIV infection. Therefore, it's an effective prevention strategy that concerns only people who are not HIV-positive. Most of the questions in this survey are about knowledge of, interest in and intention to as well as actual use of PrEP. That's why we'd kindly ask you not to fill in the questionnaire. Perhaps, you would like to suggest doing this survey to people around you?

If This questionnaire is inten... Is Displayed, Then Skip To End of Survey

Display This Question:

If What is your HIV serological status ? I don't want to provide this information Is Selected

dwtt PrEP refers to the use of antiretroviral drugs to prevent HIV infection. Therefore, it's an effective prevention strategy that concerns only people who are not HIV-positive. Most of the questions in this survey are about knowledge of, interest in and intention to as well as actual use of PrEP. If you don't want to provide your status, which we can understand, it would make analysing the data much more complicated which is why we'd kindly ask you not to fill in the questionnaire. Perhaps, you would like to suggest doing this survey to people around you?

If This questionnaire is inten... Is Displayed, Then Skip To End of Survey

prep Do you already know what PrEP is?

- Yes (1)
- No (2)

Display This Question:

If Do you already know what PrEP is? Yes Is Selected

prep_k What, from your point of view, is the best description of PrEP? (maximum 2 choices)

- PrEP is a pill that you can use after you think you have been at risk of being infected by HIV. (1)
- PrEP is a pill that greatly reduces the risk of contracting HIV. You have to take it every day. (2)
- PrEP is a pill that greatly reduces the risk of contracting HIV and Sexually Transmitted Infections (STIs). (3)
- PrEP is a pill that greatly reduces the risk of contracting HIV. You have to take it when you plan to have sex, before and two days after. (4)
- PrEP is a pill that greatly reduces the risk of contracting HIV. You have to take it once or twice a week. (5)

Display This Question:

If Do you already know what PrEP is ? Yes Is Selected

prep_s How did you learn this information? (more than one answer possible)

- From a doctor/medical personnel (1)
- From a non-governmental organisation (NGO/charity) (2)
- From one or more friends (3)
- From one or more HIV-positive persons (4)
- From the mainstream media (newspapers, Internet, etc.) (5)
- From the community media (specialised websites, blogs, etc.) (6)
- From social media (Facebook, Twitter, etc.) (7)
- From scientific papers/websites (8)
- Other (please specify) (9) ____________________

prep_information PrEP (pre-exposure prophylaxis) is the use of an HIV/antiretroviral drug by an HIV-negative individual for preventive purposes, that is, to prevent HIV infection. Truvada® is the main drug used as PrEP. Studies have shown that it can be taken on a daily basis or continuous regimen (as in PROUD or iPrex trials). Other studies have shown that it can be taken intermittently, before and after sex, on an on-demand or event-driven basis (IPERGAY study).   Currently available data show that PrEP users in both situations above are sufficiently protected against HIV if the drug is present in their blood. PrEP does not provide protection against other sexually transmitted infections (STIs). PrEP is not to be confused with PEP (post-exposure prophylaxis) which is a combination of HIV/antiretroviral drugs to prevent HIV after exposure to the virus. Requests have been made to European health authorities to permit the prescribing of Truvada® in the best possible conditions (supervision, safety, coverage and evaluation).

Prep_use Are you already using PrEP?

- Yes (1)
- No (2)

If Yes Is Selected, Then Skip To In your opinion, what would be the be...

prep_needs Please answer the following question:

|  | No, definitely not (1) | No, probably not (2) | Maybe (3) | Yes, probably (4) | Yes, definitely (5) |
| --- | --- | --- | --- | --- | --- |
| Based on your life and what you know, would PrEP meet your HIV prevention needs? (8) |  |  |  |  |  |

prep_interest About your interest in using PrEP:

|  | No, definitely not (1) | No, probably not (2) | Maybe (3) | Yes, probably (4) | Yes, definitely (5) |
| --- | --- | --- | --- | --- | --- |
| Are you interested in using PrEP? (10) |  |  |  |  |  |

conditions About your intention to use PrEP:

|  | No, definitely not (1) | No, probably not (2) | Maybe (3) | Yes, probably (4) | Yes, definitely (5) | Not applicable (6) |
| --- | --- | --- | --- | --- | --- | --- |
| Is it your intention to use it if and when it becomes officially available in your country? (conditions_1) |  |  |  |  |  |  |
| Is it your intention to use it before it becomes officially available in your country? (conditions_2) |  |  |  |  |  |  |

prep_place In your opinion, where would be the best places to prescribe PrEP? (maximum 2 choices)

- At a hospital (1)
- At a community health center, a checkpoint or LGBT/HIV clinic (2)
- At an STI clinic (3)
- At a General Practitioner's (4)
- At a NGO/charity (5)
- I don't have any preference (6)

prep_pay What is the maximum amount, if anything, that you would be willing to pay for a batch of 30 pills? (please enter only digits)

currency In which currency did you enter this amount?

- Euro (1)
- CHF (2)
- Danish Crone (3)
- Romanian Leu (4)
- GBP (5)
- Other (6) ____________________

prep_free Do you think it should be free of charge/covered by a health insurance for persons who use/need it?

- Yes (1)
- No (2)
- In part, people who use it should pay some of the cost (3)
- I don't know (4)

prep_package Please answer the following two questions:

|  | No, definitely not (1) | No, probably not (2) | Maybe (3) | Yes, probably (4) | Yes, definitely (5) |
| --- | --- | --- | --- | --- | --- |
| Do you think PrEP should be made officially available backed up by a comprehensive prevention package (regular HIV testing, STI testing and treatment, peer support, etc.)? (prep_package_1) |  |  |  |  |  |
| Would you go and get PrEP if and when such a prevention package is officially available in your country? (prep_package_2) |  |  |  |  |  |

prep_sex How would you react if a sexual partner (principal or casual) said they were on PrEP?

|  | Extremely unlikely (1) | Somewhat unlikely (2) | Neither likely nor unlikely (3) | Somewhat likely (4) | Extremely likely (5) |
| --- | --- | --- | --- | --- | --- |
| I would refuse to have sex with them (prep_sex_1) |  |  |  |  |  |
| I would ask them to use a condom (prep_sex_2) |  |  |  |  |  |
| I would have condomless sex with them (prep_sex_3) |  |  |  |  |  |

Display This Question:

If What is your interest in using PrEP? Are you interested in using PrEP? - Maybe Is Selected

Or What is your interest in using PrEP? Are you interested in using PrEP? - Yes, probably Is Selected

Or What is your interest in using PrEP? Are you interested in using PrEP? - Yes, definitely Is Selected

Prep_int_r1 Why are you interested in taking PrEP?

|  | Strongly disagree (1) | Somewhat disagree (2) | Neither agree or disagree (3) | Somewhat agree (4) | Strongly agree (5) |
| --- | --- | --- | --- | --- | --- |
| I'd rather have condomless sex (Prep_int_r1_1) |  |  |  |  |  |
| I'm at risk of being infected by HIV (Prep_int_r1_2) |  |  |  |  |  |
| I would feel safer (Prep_int_r1_3) |  |  |  |  |  |
| I would feel less anxious (Prep_int_r1_4) |  |  |  |  |  |
| I would feel more in control (Prep_int_r1_5) |  |  |  |  |  |
| I would have a more satisfying sex life (Prep_int_r1_6) |  |  |  |  |  |

Display This Question:

If What is your interest in using PrEP? Are you interested in using PrEP? - Maybe Is Selected

Or What is your interest in using PrEP? Are you interested in using PrEP? - Yes, probably Is Selected

Or What is your interest in using PrEP? Are you interested in using PrEP? - Yes, definitely Is Selected

prep_int_r2 Would you still be interested in taking PrEP...

|  | Extremely unlikely (1) | Somewhat unlikely (2) | Neither likely nor unlikely (3) | Somewhat likely (4) | Extremely likely (5) |
| --- | --- | --- | --- | --- | --- |
| Even if you had to pay for it? (prep_int_r2_1) |  |  |  |  |  |
| Even if it meant taking medication everyday? (prep_int_r2_2) |  |  |  |  |  |
| Even though there are side-effects? (prep_int_r2_3) |  |  |  |  |  |
| Even if you have to undergo regular medical check-ups? (prep_int_r2_4) |  |  |  |  |  |
| Even if you had to go to the hospital to get it? (prep_int_r2_5) |  |  |  |  |  |

Display This Question:

If What is your interest in using PrEP? Are you interested in using PrEP? - No, definitely not Is Selected

Or What is your interest in using PrEP? Are you interested in using PrEP? - No, probably not Is Selected

prep_int_not Why are you not interested in taking PrEP?

|  | Strongly disagree (1) | Somewhat disagree (2) | Neither agree nor disagree (3) | Somewhat agree (4) | Strongly agree (5) |
| --- | --- | --- | --- | --- | --- |
| I don't want to take medication every day (prep_int_not_1) |  |  |  |  |  |
| I don't want to pay for PrEP (prep_int_not_2) |  |  |  |  |  |
| I'm worried about the side-effects (prep_int_not_3) |  |  |  |  |  |
| I'm afraid of being seen in a negative light if I take PrEP (prep_int_not_4) |  |  |  |  |  |
| I don't believe it works (prep_int_not_5) |  |  |  |  |  |
| I'm worried of getting other STIs (prep_int_not_6) |  |  |  |  |  |
| I don't need to change how I protect myself (prep_int_not_7) |  |  |  |  |  |
| I don't think I'm at risk of being infected by HIV (prep_int_not_8) |  |  |  |  |  |
| I don't want to undergo regular medical check-ups (prep_int_not_9) |  |  |  |  |  |
| I'm worried I might use condoms less often (prep_int_not_10) |  |  |  |  |  |

gender_birth Your gender at birth was:

- Male (1)
- Female (2)
- Other (3)
- I prefer not to say (4)

gender_now Your gender now is:

- Male (1)
- Female (2)
- Non-binary/other (3)
- I prefer not to say (4)

age How old are you?

country What country were you born in?

- Afghanistan (1)
- Albania (2)
- Algeria (3)
- Andorra (4)
- Angola (5)
- Antigua and Barbuda (6)
- Argentina (7)
- Armenia (8)
- Australia (9)
- Austria (10)
- Azerbaijan (11)
- Bahamas (12)
- Bahrain (13)
- Bangladesh (14)
- Barbados (15)
- Belarus (16)
- Belgium (17)
- Belize (18)
- Benin (19)
- Bhutan (20)
- Bolivia (21)
- Bosnia and Herzegovina (22)
- Botswana (23)
- Brazil (24)
- Brunei Darussalam (25)
- Bulgaria (26)
- Burkina Faso (27)
- Burundi (28)
- Cambodia (29)
- Cameroon (30)
- Canada (31)
- Cape Verde (32)
- Central African Republic (33)
- Chad (34)
- Chile (35)
- China (36)
- Colombia (37)
- Comoros (38)
- Congo, Republic of the... (39)
- Costa Rica (40)
- Côte d'Ivoire (41)
- Croatia (42)
- Cuba (43)
- Cyprus (44)
- Czech Republic (45)
- Democratic Republic of the Congo (46)
- Denmark (47)
- Djibouti (48)
- Dominica (49)
- Dominican Republic (50)
- Ecuador (51)
- Egypt (52)
- El Salvador (53)
- Equatorial Guinea (54)
- Eritrea (55)
- Estonia (56)
- Ethiopia (57)
- Fiji (58)
- Finland (59)
- France (60)
- Gabon (61)
- Gambia (62)
- Georgia (63)
- Germany (64)
- Ghana (65)
- Greece (66)
- Grenada (67)
- Guatemala (68)
- Guinea (69)
- Guinea-Bissau (70)
- Guyana (71)
- Haiti (72)
- Honduras (73)
- Hong Kong (S.A.R.) (74)
- Hungary (75)
- Iceland (76)
- India (77)
- Indonesia (78)
- Iran, Islamic Republic of... (79)
- Iraq (80)
- Ireland (81)
- Israel (82)
- Italy (83)
- Jamaica (84)
- Japan (85)
- Jordan (86)
- Kazakhstan (87)
- Kenya (88)
- Kiribati (89)
- Kuwait (90)
- Kyrgyzstan (91)
- Lao People's Democratic Republic (92)
- Latvia (93)
- Lebanon (94)
- Lesotho (95)
- Liberia (96)
- Libyan Arab Jamahiriya (97)
- Liechtenstein (98)
- Lithuania (99)
- Luxembourg (100)
- Madagascar (101)
- Malawi (102)
- Malaysia (103)
- Maldives (104)
- Mali (105)
- Malta (106)
- Marshall Islands (107)
- Mauritania (108)
- Mauritius (109)
- Mexico (110)
- Micronesia, Federated States of... (111)
- Monaco (112)
- Mongolia (113)
- Montenegro (114)
- Morocco (115)
- Mozambique (116)
- Myanmar (117)
- Namibia (118)
- Nauru (119)
- Nepal (120)
- Netherlands (121)
- New Zealand (122)
- Nicaragua (123)
- Niger (124)
- Nigeria (125)
- North Korea (126)
- Norway (127)
- Oman (128)
- Pakistan (129)
- Palau (130)
- Panama (131)
- Papua New Guinea (132)
- Paraguay (133)
- Peru (134)
- Philippines (135)
- Poland (136)
- Portugal (137)
- Qatar (138)
- Republic of Moldova (139)
- Romania (140)
- Russian Federation (141)
- Rwanda (142)
- Saint Kitts and Nevis (143)
- Saint Lucia (144)
- Saint Vincent and the Grenadines (145)
- Samoa (146)
- San Marino (147)
- Sao Tome and Principe (148)
- Saudi Arabia (149)
- Senegal (150)
- Serbia (151)
- Seychelles (152)
- Sierra Leone (153)
- Singapore (154)
- Slovakia (155)
- Slovenia (156)
- Solomon Islands (157)
- Somalia (158)
- South Africa (159)
- South Korea (160)
- Spain (161)
- Sri Lanka (162)
- Sudan (163)
- Suriname (164)
- Swaziland (165)
- Sweden (166)
- Switzerland (167)
- Syrian Arab Republic (168)
- Tajikistan (169)
- Thailand (170)
- The former Yugoslav Republic of Macedonia (171)
- Timor-Leste (172)
- Togo (173)
- Tonga (174)
- Trinidad and Tobago (175)
- Tunisia (176)
- Turkey (177)
- Turkmenistan (178)
- Tuvalu (179)
- Uganda (180)
- Ukraine (181)
- United Arab Emirates (182)
- United Kingdom of Great Britain and Northern Ireland (183)
- United Republic of Tanzania (184)
- United States of America (185)
- Uruguay (186)
- Uzbekistan (187)
- Vanuatu (188)
- Venezuela, Bolivarian Republic of... (189)
- Viet Nam (190)
- Yemen (191)
- Zambia (192)
- Zimbabwe (193)

residence In which country do you live at the moment?

- Denmark (1)
- United Kingdom (2)
- France (3)
- Germany (4)
- Greece (5)
- Netherlands (6)
- Italy (7)
- Portugal (8)
- Romania (9)
- Spain (10)
- Switzerland (11)
- Ireland (12)
- Other (13)

Display This Question:

If List of Countries Other Is Selected

residence_other In which country do you live at the moment?

- Afghanistan (1)
- Albania (2)
- Algeria (3)
- Andorra (4)
- Angola (5)
- Antigua and Barbuda (6)
- Argentina (7)
- Armenia (8)
- Australia (9)
- Austria (10)
- Azerbaijan (11)
- Bahamas (12)
- Bahrain (13)
- Bangladesh (14)
- Barbados (15)
- Belarus (16)
- Belgium (17)
- Belize (18)
- Benin (19)
- Bhutan (20)
- Bolivia (21)
- Bosnia and Herzegovina (22)
- Botswana (23)
- Brazil (24)
- Brunei Darussalam (25)
- Bulgaria (26)
- Burkina Faso (27)
- Burundi (28)
- Cambodia (29)
- Cameroon (30)
- Canada (31)
- Cape Verde (32)
- Central African Republic (33)
- Chad (34)
- Chile (35)
- China (36)
- Colombia (37)
- Comoros (38)
- Congo, Republic of the... (39)
- Costa Rica (40)
- Côte d'Ivoire (41)
- Croatia (42)
- Cuba (43)
- Cyprus (44)
- Czech Republic (45)
- Democratic Republic of the Congo (46)
- Denmark (47)
- Djibouti (48)
- Dominica (49)
- Dominican Republic (50)
- Ecuador (51)
- Egypt (52)
- El Salvador (53)
- Equatorial Guinea (54)
- Eritrea (55)
- Estonia (56)
- Ethiopia (57)
- Fiji (58)
- Finland (59)
- France (60)
- Gabon (61)
- Gambia (62)
- Georgia (63)
- Germany (64)
- Ghana (65)
- Greece (66)
- Grenada (67)
- Guatemala (68)
- Guinea (69)
- Guinea-Bissau (70)
- Guyana (71)
- Haiti (72)
- Honduras (73)
- Hong Kong (S.A.R.) (74)
- Hungary (75)
- Iceland (76)
- India (77)
- Indonesia (78)
- Iran, Islamic Republic of... (79)
- Iraq (80)
- Ireland (81)
- Israel (82)
- Italy (83)
- Jamaica (84)
- Japan (85)
- Jordan (86)
- Kazakhstan (87)
- Kenya (88)
- Kiribati (89)
- Kuwait (90)
- Kyrgyzstan (91)
- Lao People's Democratic Republic (92)
- Latvia (93)
- Lebanon (94)
- Lesotho (95)
- Liberia (96)
- Libyan Arab Jamahiriya (97)
- Liechtenstein (98)
- Lithuania (99)
- Luxembourg (100)
- Madagascar (101)
- Malawi (102)
- Malaysia (103)
- Maldives (104)
- Mali (105)
- Malta (106)
- Marshall Islands (107)
- Mauritania (108)
- Mauritius (109)
- Mexico (110)
- Micronesia, Federated States of... (111)
- Monaco (112)
- Mongolia (113)
- Montenegro (114)
- Morocco (115)
- Mozambique (116)
- Myanmar (117)
- Namibia (118)
- Nauru (119)
- Nepal (120)
- Netherlands (121)
- New Zealand (122)
- Nicaragua (123)
- Niger (124)
- Nigeria (125)
- North Korea (126)
- Norway (127)
- Oman (128)
- Pakistan (129)
- Palau (130)
- Panama (131)
- Papua New Guinea (132)
- Paraguay (133)
- Peru (134)
- Philippines (135)
- Poland (136)
- Portugal (137)
- Qatar (138)
- Republic of Moldova (139)
- Romania (140)
- Russian Federation (141)
- Rwanda (142)
- Saint Kitts and Nevis (143)
- Saint Lucia (144)
- Saint Vincent and the Grenadines (145)
- Samoa (146)
- San Marino (147)
- Sao Tome and Principe (148)
- Saudi Arabia (149)
- Senegal (150)
- Serbia (151)
- Seychelles (152)
- Sierra Leone (153)
- Singapore (154)
- Slovakia (155)
- Slovenia (156)
- Solomon Islands (157)
- Somalia (158)
- South Africa (159)
- South Korea (160)
- Spain (161)
- Sri Lanka (162)
- Sudan (163)
- Suriname (164)
- Swaziland (165)
- Sweden (166)
- Switzerland (167)
- Syrian Arab Republic (168)
- Tajikistan (169)
- Thailand (170)
- The former Yugoslav Republic of Macedonia (171)
- Timor-Leste (172)
- Togo (173)
- Tonga (174)
- Trinidad and Tobago (175)
- Tunisia (176)
- Turkey (177)
- Turkmenistan (178)
- Tuvalu (179)
- Uganda (180)
- Ukraine (181)
- United Arab Emirates (182)
- United Kingdom of Great Britain and Northern Ireland (183)
- United Republic of Tanzania (184)
- United States of America (185)
- Uruguay (186)
- Uzbekistan (187)
- Vanuatu (188)
- Venezuela, Bolivarian Republic of... (189)
- Viet Nam (190)
- Yemen (191)
- Zambia (192)
- Zimbabwe (193)

location At the moment, you live in:

- A very large city (population of 1 million or more) (1)
- A large city (population of 500,000 to 1 million) (2)
- A medium-sized city (population of 100,000 to 500,000) (3)
- A small city (population of 10,000 to 100,000) (4)
- A town (population under 10,000) (5)

relationship What is your current relationship status?

- Single (1)
- Having dates (2)
- In a relationship (3)
- In an open relationship (4)

children Do you have any children?

- Yes (1)
- No (2)

school_y How many years did you go to school?

- 0-9 years (1)
- 10 years or more (2)

edu_h Are you attending or have you finished higher education?

- Yes (1)
- No (2)

Display This Question:

If What is the highest qualification you have obtained or what studies are you currently enrolled in? Professional/Vocational qualification Is Selected

edu_d What studies are you currently enrolled in or what is the highest qualification you obtained?

- Professional/Vocational qualification (1)
- Bachelor's degree or equivalent (2)
- Master's degree or equivalent (3)
- PhD/Doctorate or equivalent (4)

financial Currently, how would you say you are doing financially?

- You can't make ends meet without borrowing (1)
- You are having problems making ends meet (2)
- You are getting by but have to be careful (3)
- Things are all right (4)
- You are doing rather well (5)
- You are doing really well (6)

satisf How satisfied are you?

|  | Extremely dissatisfied (1) | Somewhat dissatisfied (2) | Neither satisfied nor dissatisfied (3) | Somewhat satisfied (4) | Extremely satisfied (5) |
| --- | --- | --- | --- | --- | --- |
| Considering your life in general, would you say that you are: (satisf_1) |  |  |  |  |  |
| Concerning your sex life, would you say that you are: (satisf_2) |  |  |  |  |  |

sex_ever Have you had vaginal and/or anal sex?

- Yes, in the past 6 months (1)
- Yes, more than 6 months ago (2)
- No, never (3)

Display This Question:

If Have you ever had sex ? Yes, in the past 12 months Is Selected

Or Have you ever had sex ? Yes, more than a year ago Is Selected

first_time How old were you when you first had sex? Please enter a number only, for example 15.

Display This Question:

If Have you ever had sex ? Yes, in the past 12 months Is Selected

sex_whom In the past 6 months, you have had sex with: (more than one answer possible)

- Men (1)
- Women (2)
- Transgenders/Transsexuals (3)

Hiv_risk Currently, how do you rate your risk of becoming infected with....?

|  | Low (1) | Rather low (2) | Average (3) | Rather high (4) | High (5) |
| --- | --- | --- | --- | --- | --- |
| HIV (Hiv_risk_1) |  |  |  |  |  |
| An STI (gonorrhea, chlamydia, syphilis, etc.) (Hiv_risk_2) |  |  |  |  |  |

Display This Question:

If Have you ever had sex ? Yes, in the past 12 months Is Selected

main_partner Do you have a main sex partner (e.g. boyfriend/girlfriend or husband/wife) at the moment?

- Yes (1)
- No (2)

Display This Question:

If Do you have a main sex partner at the moment ? Yes Is Selected

main_partner_hiv Your main sex partner is:

- HIV-negative (1)
- HIV-positive (2)
- I don't know their HIV status (3)
- I don't want to say it (4)

Display This Question:

If Your main partner is : HIV-positive Is Selected

main_partner_vl Do you know what the viral load of your main sex partner is?

- I don't know what a viral load is (1)
- Yes, their viral load is detectable (2)
- Yes, their viral load is undetectable (3)
- No, I don't know what their viral load is (4)

Display This Question:

If Do you have a main sex partner (e.g. boyfriend/girlfriend or husband/wife) at the moment? Yes Is Selected

partners In the past 6 months, have you had sex with partners other than your main sex partner?

- Yes (1)
- No (2)

Display This Question:

If In the past 6 months, have you had sex with partners other than your main sex partner? Yes Is Selected

casual Were they:

- One-off, casual encounters (1)
- Regular sex partners (e.g. fuck buddies or sex friends) (2)

Display This Question:

If Do you have a main sex partner (e.g. boyfriend/girlfriend or husband/wife) at the moment? No Is Selected

Or In the past 6 months, have you had sex with partners other than your main sex partner? Yes Is Selected

n_sex_p How many different sex partners have you had in the past 6 months, excluding your main partner, if you have one? [Give an approximate figure if you don't know the exact number]

Display This Question:

If Have you ever had sex ? Yes, in the past 12 months Is Selected

freq_sex In the past 6 months, how often did you have:

|  | Never (1) | Less than once a month (2) | Once a month or more (3) | Once a week or more (4) | Daily (5) |
| --- | --- | --- | --- | --- | --- |
| Vaginal sex (freq_sex_1) |  |  |  |  |  |
| Anal sex (freq_sex_2) |  |  |  |  |  |

Display This Question:

If Do you have a main sex partner (e.g. boyfriend/girlfriend or husband/wife) at the moment? No Is Selected

Or In the past 6 months, have you had sex with partners other than your main sex partner? Yes Is Selected

risk_red In the past 6 months, excluding your main partner (if you have one), how often have you used the following methods with these partners to reduce the risk of HIV infection?

|  | Never (1) | Rarely (2) | From time to time (3) | Nearly always (4) | Always (5) | Not applicable (6) |
| --- | --- | --- | --- | --- | --- | --- |
| I use a condom for vaginal sex (risk_red_1) |  |  |  |  |  |  |
| I use a condom for anal sex (risk_red_2) |  |  |  |  |  |  |
| I choose partners who say they are HIV-negative (risk_red_3) |  |  |  |  |  |  |
| I adapt the sex I have depending on my partner's HIV-status (risk_red_4) |  |  |  |  |  |  |
| I have sex with HIV-positive partners who say their viral load is undetectable (risk_red_5) |  |  |  |  |  |  |

Display This Question:

If Have you ever had sex ? Yes, in the past 12 months Is Selected

Or Have you ever had sex ? Yes, more than a year ago Is Selected

cond_inter Did you use a condom when you last had vaginal and/or anal sex?

- Yes (1)
- No (2)

Display This Question:

If Have you ever had sex ? Yes, in the past 12 months Is Selected

Or Have you ever had sex ? Yes, more than a year ago Is Selected

sex_work Have you ever received money, goods or drugs in exchange for sex?

- Yes, in the past 12 months (1)
- Yes, more than a year ago (2)
- No, never (3)

drug_i Have you ever injected yourself or been injected with drugs?

- Yes, in the past 12 months (1)
- Yes, more than a year ago (2)
- No, never (3)

Display This Question:

If Have you ever had sex ? Yes, in the past 12 months Is Selected

Or Have you ever had sex ? Yes, more than a year ago Is Selected

And If

If Have you ever injected yourself or been injected with recreational drugs ? Yes, more than a year ago Is Selected

Or Have you ever injected yourself or been injected with recreational drugs ? Yes, in the past 12 months Is Selected

chem_sex_i Did you inject or were you injected with drugs in a sexual context? (e.g. slam)

- Yes (1)
- No (2)

drug_o Have you ever taken drugs (other than by injection)?

- Yes, in the past 12 months (1)
- Yes, more than a year ago (2)
- No, never (3)

Display This Question:

If Have you ever had sex ? Yes, in the past 12 months Is Selected

Or Have you ever had sex ? Yes, more than a year ago Is Selected

And If

If Have you ever taken recreational drugs (other than by injection) ? Yes, in the past 12 months Is Selected

Or Have you ever taken recreational drugs (other than by injection) ? Yes, more than a year ago Is Selected

chem_sex_o Did you take drugs in a sexual context? (e.g. chemsex parties, smoking crystal meth etc.)

- Yes (1)
- No (2)

Display This Question:

If Have you ever had sex ? Yes, in the past 12 months Is Selected

Or Have you ever had sex ? Yes, more than a year ago Is Selected

abuse Have you ever had sex against your will because of verbal, physical or any other form of pressure?

- Yes (1)
- No (2)

hiv Have you ever been tested for HIV?

- Yes, in the past 12 months (1)
- Yes, more than a year ago (2)
- No, never (3)

Display This Question:

If Have you ever been tested for HIV ? Yes, in the past 12 months Is Selected

hiv_freq How many HIV tests have you had in the past 12 months?

- 1 (1)
- 2 (2)
- 3 (3)
- 4 (4)
- 5 or more (5)

Display This Question:

If Have you ever had sex ? No, never Is Selected

end This is the end of the questionnaire. Thank you very much for taking part in the survey. If you have any comments, feedback or wish to add any additional information, please fill in the box below.

If This is the end of the ques... Is Displayed, Then Skip To End of Survey

STI Have you ever been diagnosed with a sexually transmitted infection (other than HIV)?

- Yes, in the past 12 months (1)
- Yes, more than a year ago (2)
- No, never (3)
- I don't know (4)

Display This Question:

If Have you ever been diagnosed with a sexually transmitted infection (other than HIV)? Yes, in the past 12 months Is Selected

STI_freq How many times have you been diagnosed with an STI in the past 12 months?

- 1 (1)
- 2 (2)
- 3 (3)
- 4 (4)
- 5 or more (5)

pep Have you ever used post-exposure prophylaxis for HIV (PEP or "emergency treatment") directly after being exposed to HIV? (and you didn't use it as PrEP)

- Yes, more than once during the past 12 months (1)
- Yes, once during the past 12 months (2)
- Yes, more than a year ago (3)
- No, never (4)

prep_study At the moment, are you participating in a study in which PrEP is provided?

- Yes (1)
- No (2)

Display This Question:

If At the moment, are you participating in a study in which PrEP is provided ? Yes Is Selected

end2 This is the end of the questionnaire. Thank you very much for taking part in the survey. If you have any comments, feedback or wish to add any additional information, please fill in the box below.

If This is the end of the ques... Is Displayed, Then Skip To End of Survey

hiv_treat Have you ever taken medication (PrEP) to prevent an HIV infection?

- Yes (1)
- No (2)

Display This Question:

If Have you ever taken an HIV treatment before sex to prevent transmission ? No Is Selected

end3 This is the end of the questionnaire. Thank you very much for taking part in the survey. If you have any comments, feedback or wish to add any additional information, please fill in the box below.

If This is the end of the ques... Is Displayed, Then Skip To End of Survey

prep_use_t For how many months have you been using PrEP?

- I'm no longer using it at the moment (1)
- Less than 3 months (2)
- 3-6 months (3)
- 7-12 months (4)
- For more than a year (please indicate length of time): (5) ____________________

regimen What tablet dosing regimen do you (or did you) usually use ?

- Every day (continuous regimen) (1)
- Before and after sex or on-demand (intermittent regimen) (2)
- Other dosing regimen (please specify): (3) ____________________

type_prep Which drug are you using or did you use for PrEP ? (more than one answer possible)

- Truvada® (1)
- Generic Truvada® (2)
- Other (please specify): (3) ____________________

Display This Question:

If Which drug are you using or did you use for PrEP ? (more than one answer possible) Other (please specify): Is Selected

other_prep Why did you choose this drug (other than Truvada® or generic Truvada®)? (2 answers maximum)

- You trust the effectiveness of (all) HIV treatments (1)
- You use Truvada® in general and supplement from time to time with other medication (2)
- You only have access to other medication (3)
- Other (please explain): (4) ____________________

prep_year What year did you take PrEP for the first time? (for example: 2012)

prep_why When you took PrEP for the first time, it was:

- After you made some inquiries yourself (1)
- After a doctor or a health care worker told you about it (2)
- After a friend and/or a partner told you about it (3)
- After you heard about it from a NGO/charity (4)
- Other (please specify) (5) ____________________

prep_miss Have you ever skipped a dose or stopped temporarily?

- Yes (1)
- No (2)

Display This Question:

If Have you ever skipped a dose or stopped temporarily? Yes Is Selected

And What tablet dosing regimen do you (or did you) usually use ? Every day (continuous regimen) Is Selected

prep_miss_alt In the past 3 months, how many times and for how long did you take breaks? (For example, if you stopped once for 4 days and twice for 6 days, click in the row "4 days" and "6 days" and enter "once" with "4 days" and "2-4 times"  under "6 days")

|  | Never (1) | Once (2) | 2-4 times (3) | 5-7 times (4) | More than 8 times (5) |
| --- | --- | --- | --- | --- | --- |
| 1 day (prep_miss_alt_1) |  |  |  |  |  |
| 2 days (prep_miss_alt_2) |  |  |  |  |  |
| 3 days (prep_miss_alt_3) |  |  |  |  |  |
| 4 days (prep_miss_alt_4) |  |  |  |  |  |
| 5 days (prep_miss_alt_5) |  |  |  |  |  |
| 6 days (prep_miss_alt_6) |  |  |  |  |  |
| 7 days (prep_miss_alt_7) |  |  |  |  |  |
| >7 days (prep_miss_alt_8) |  |  |  |  |  |

Display This Question:

If If you're taking/used to take PrEP on a daily basis, have you or did you ever miss a dose or stop temporarily ? Yes Is Selected

prep_miss_r Think about the last break in your PrEP. What were the main reasons? (3 answers maximum)

- I forgot to take my tablets (1)
- I ran out of tablets (2)
- I was no longer having sex (3)
- I changed partners (4)
- I experienced side effects (5)
- I was no longer with my main partner (6)
- I preferred other prevention tools (condoms, regular testing, choosing partners with an undetectable viral load, etc.) (7)
- I could not afford to get PrEP anymore (8)
- I could not obtain PrEP anymore (9)
- Other (please explain): (10) ____________________

Display This Question:

If What tablet dosing regimen do you (or did you) usually use ? Around the time of sexual relations (intermittent regimen) Is Selected

Or What tablet dosing regimen do you (or did you) usually use ? Other dosing regiment (please specify): Is Selected

prep_inter_freq How often do you take PrEP?

- Several times a week (1)
- Once or twice a week (2)
- Less than once a week (3)
- Once a month (4)
- Less than once a month (5)

Display This Question:

If What tablet dosing regimen do you (or did you) usually use ? Before and after sex or on-demand (intermittent regimen) Is Selected

Or What tablet dosing regimen do you (or did you) usually use ? Other dosing regimen (please specify): Is Selected

prep_inter_when You take PrEP:

- Every time you have sex (1)
- Just when you think it's risky (2)

prep_source In general, how have you obtained your PrEP tablets? (more than one answer possible)

- With a medical prescription from a doctor (paying myself) (1)
- With a medical prescription for curative use (you said that you were HIV-positive) (2)
- By using HIV treatments prescribed as post-exposure prophylaxis (PEP) (you said that you were HIV-negative and you had a risky behaviour) (3)
- From a NGO/charity (4)
- An HIV-positive friend gives/gave you their HIV treatment (5)
- From a participant in a PrEP clinical trial (6)
- From another PrEP user (outside of a clinical trial) (7)
- By purchasing them online (8)
- By purchasing generics in other countries of the world (9)
- By buying them in the street, at leisure-time/entertainment or sexual venues (10)
- Other (please specify) (11) ____________________

prep_costs On average, on a monthly basis, how much do/did you spend on PrEP?

currency2 In which currency did you enter the amount?

- Euro (1)
- CHF (2)
- Danish Crone (3)
- Romanian Leu (4)
- GBP (5)
- Other (please specify) (6) ____________________

prep_income What percentage of your monthly income does this amount represent?

- (1)
- 5-10% (2)
- 10-25% (3)
- 25-50% (4)
- >50% (5)

prep_public Have you told those around you that you are taking PrEP?

|  | Yes (1) | No (2) | Not applicable (3) |
| --- | --- | --- | --- |
| Friends (prep_public_1) |  |  |  |
| HIV-positive friends (prep_public_2) |  |  |  |
| Family (prep_public_3) |  |  |  |
| Main sex partner (prep_public_4) |  |  |  |
| Occasional sex partners (prep_public_5) |  |  |  |
| Doctors or other medical personnel (prep_public_6) |  |  |  |
| Members of a NGO/charity (prep_public_7) |  |  |  |
| I publicly display it on dating sites/apps (prep_public_8) |  |  |  |
| I advocate for PrEP (I tell everyone around me that I'm using it) (prep_public_9) |  |  |  |

prep_checks Have you had any medical check-ups specifically related to your taking PrEP (for example, tests for your kidneys)?

- Yes (1)
- No (2)

Display This Question:

If Have you had any medical check-ups specifically related to your taking PrEP (for example, tests f... Yes Is Selected

prep_check_fre How often do/did you get medical check-ups with regard to PrEP?

- Only once, before I started (1)
- Every 3 months (2)
- Irregularly (3)

prep_crea Are you taking products to increase your muscle mass and performance?

- Yes, creatine products (1)
- Yes, protein (whey) (2)
- Yes, both creatine and protein (whey) (3)
- No (4)

prep_condom2 When you are on PrEP, do you accept that your partners want to use a condom?

- Never (1)
- Rarely (2)
- From time to time (3)
- Nearly always (4)
- Always (5)

prep_hiv Since you started taking PrEP, would you say that...

|  | A lot lower than before (1) | Lower than before (2) | The same (3) | Higher than before (4) | A lot higher than before (5) |
| --- | --- | --- | --- | --- | --- |
| Your condom use is: (prep_hiv_1) |  |  |  |  |  |
| Your frequency of HIV testing: (prep_hiv_2) |  |  |  |  |  |
| Your risk of infection from HIV: (prep_hiv_3) |  |  |  |  |  |

prep_qol Since you started taking PrEP, would you say that:

|  | A lot worse (1) | Worse (2) | The same (3) | Better (4) | A lot better (5) |
| --- | --- | --- | --- | --- | --- |
| Your quality of life in general is: (prep_qol_1) |  |  |  |  |  |
| The quality of your sex life is: (prep_qol_2) |  |  |  |  |  |

END_4 This is the end of the questionnaire. Thank you very much for taking part in the survey. If you have any comments, feedback or wish to add any additional information, please fill in the box below.

If This is the end of the ques... Is Displayed, Then Skip To End of Survey
